# CRL4 antagonizes SCF^Fbxo7^-mediated turnover of cereblon and BK channel to regulate learning and memory

**DOI:** 10.1371/journal.pgen.1007165

**Published:** 2018-01-25

**Authors:** Tianyu Song, Shenghui Liang, Jiye Liu, Tingyue Zhang, Yifei Yin, Chenlu Geng, Shaobing Gao, Yan Feng, Hao Xu, Dongqing Guo, Amanda Roberts, Yuchun Gu, Yong Cang

**Affiliations:** 1 Life Sciences Institute and Innovation Center for Cell Signalling Network, Zhejiang University, Hangzhou, Zhejiang, China; 2 Translational and Regenerative Medicine Center, Aston Medical School, Aston University, Birmingham, United Kingdom; 3 Laboratory of Molecular Pharmacology, Institute of Molecular Medicine, Peking University, Peking, China; 4 Molecular and Cellular Neurosciences Department, The Scripps Research Institute, University of California, San Diego, La Jolla, California, United States of America; 5 School of Life Science and Technology, ShanghaiTech University, Shanghai, China; Florey Institute of Neuroscience and Mental Health, AUSTRALIA

## Abstract

Intellectual disability (ID), one of the most common human developmental disorders, can be caused by genetic mutations in Cullin 4B (Cul4B) and cereblon (CRBN). CRBN is a substrate receptor for the Cul4A/B-DDB1 ubiquitin ligase (CRL4) and can target voltage- and calcium-activated BK channel for ER retention. Here we report that ID-associated CRL4^CRBN^ mutations abolish the interaction of the BK channel with CRL4, and redirect the BK channel to the SCF^Fbxo7^ ubiquitin ligase for proteasomal degradation. Glioma cell lines harbouring CRBN mutations record density-dependent decrease of BK currents, which can be restored by blocking Cullin ubiquitin ligase activity. Importantly, mice with neuron-specific deletion of DDB1 or CRBN express reduced BK protein levels in the brain, and exhibit similar impairment in learning and memory, a deficit that can be partially rescued by activating the BK channel. Our results reveal a competitive targeting of the BK channel by two ubiquitin ligases to achieve exquisite control of its stability, and support changes in neuronal excitability as a common pathogenic mechanism underlying CRL4^CRBN^–associated ID.

## Author summary

Our study first demonstrates intellectual disability associated proteins, CRBN and BK channel pore-forming α subunit, are targeted by SCF^Fbxo7^ for ubiquitination and proteolysis, which can be further regulated by their binding to DDB1 and the expression of CRL4 complex. We also first find DDB1 or CRBN deletion in mouse brain causes similar spatial cognitive defects. By cellular electrophysiology, human ID-related mutation and genetic mouse model experiments, we confirm the role of this regulatory pathway in controlling BK channel activity and modulating learning and memory.

## Introduction

Intellectual disability (ID), formerly known as mental retardation (MR), is a generalized neurodevelopmental disorder defined as substantial impairment of cognitive and adaptive functions with a diagnosis of IQ score of less than 70 [1]. ID can occur as an isolated phenomenon or may frequently be accompanied by congenital malformations or other neurological features such as sensory impairment, seizures, and autism spectrum disorders (ASD) [2]. With an estimated 1% of the world’s population affected [3], ID has become a major social problem and resulted in an enormous economic burden in all countries [4,5].

Many inheritable genetic mutations have been identified in ID patients [5–8], but the underlying mechanisms remain to be elucidated [9]. *Cul4B* was identified as one of the most frequently mutated genes in X-linked ID families by whole-exon sequencing [10–12]. Cul4B shares 80% sequence homology with Cul4A and they are functionally redundant [13]. Cul4A/B functions as a scaffold to bridge the RING finger protein Rbx1/Roc1 and the adaptor protein Damaged DNA Binding protein 1 (DDB1) to form the E3 ubiquitin ligase complex, Cul4 RING ligase (CRL4) [14]. CRL4 targets diverse substrates to regulate cell cycle, DNA damage repair, and chromatin functions via substrate receptors called DDB1 and Cul4 associated factors (DCAFs) [13,15,16].

Cereblon (CRBN), a DCAF protein, can recruit two ion channels to CRL4, the ClC-1 chloride channel for turnover [17] and the BK channel for trafficking [18]. Based on the study designed to overexpress CRBN and BK channel in heterologous cells, wild-type (WT) CRBN can restrict excessive BK channel in ER for a balance of BK activity in cell membrane to prevent induced epileptogenesis [18]. However, whether genetic inactivation of CRBN can influence endogenous BK channel activity need further clearly addressed.

Mutations in both CRBN and BK channel have been implicated in the pathogenesis of ID. BK channel regulates the general excitability of neurons and the transmitter release at presynaptic terminals [19]. Some ID patients with ASD and epilepsy were found to harbor a loss-of-function mutation in α subunit (*SLO1*) of BK channel [20]. Inactivation of BK channel by transcriptional silencing of the *Fmr1* gene causes the most common inheritable ID, known as Fragile X syndrome (FXS) [21,22]. Autosomal recessive CRBN mutations have also been identified in ID patients in two unrelated families [23,24]. Genetic deletion of *Slo1* [25] or *Crbn* [26] in mouse brain leads to impaired spatial learning or conditioned fear memory, respectively. Given our recent finding that CRL4^CRBN^ ubiquitinates Slo1 [18], these human and animal studies suggest that the ubiquitination of BK channel may play an important role in learning and memory.

Here using DDB1 and CRBN mutant mouse strains, we found similar cognitive defects in them and by surprise found that the total BK channel level is significantly reduced in these mutant mice brains. We further identified the ID-associated CRL4^CRBN^ mutations redirect both CRBN and BK channel to another new Cullin E3 ligase, SCF^Fbxo7^, for ubiquitination and turnover. We finally demonstrated that BK channel activity is critical for restoring the learning and memory defects observed in CRL4^CRBN^-deficient mice. Thus, our study offers a new regulatory point to control BK channel activity via two distinct ubiquitin ligase, which can be targeted for mitigating intellectual disability.

## Results

### DDB1 deletion in mouse brain leads to cognitive defects

To investigate the role of CRL4 in cognitive function, we generated the *Ddb1*^*F/F*^*;Camk2α-Cre* mouse strain, in which DDB1 is conditionally deleted in postnatal neurons in the hippocampus and cerebral cortex [18]. Whole-brain deletion of DDB1 causes neonatal death in *Ddb1*^*F/F*^*;Nestin-Cre* mice [27]. We first assessed the general locomotor and visual capabilities of the *Ddb1*^*F/F*^*;Camk2α-Cre* mice. There was no statistically significant difference between WT and *Ddb1*^*F/F*^*;Camk2α-Cre* mice in ambulation, rearing, center and total horizontal activities (S1A Fig). In a light/dark transfer test, *Ddb1*^*F/F*^*;Camk2α-Cre* mice exhibited slightly more frequent light/dark transitions as a sign of mild anxiety but no obvious visual disability (S1B Fig), the latter corroborated by another vision test (S1C Fig). This model therefore would allow us to perform cognitive tests that rely on functional locomotion and vision.

**Fig 1:**
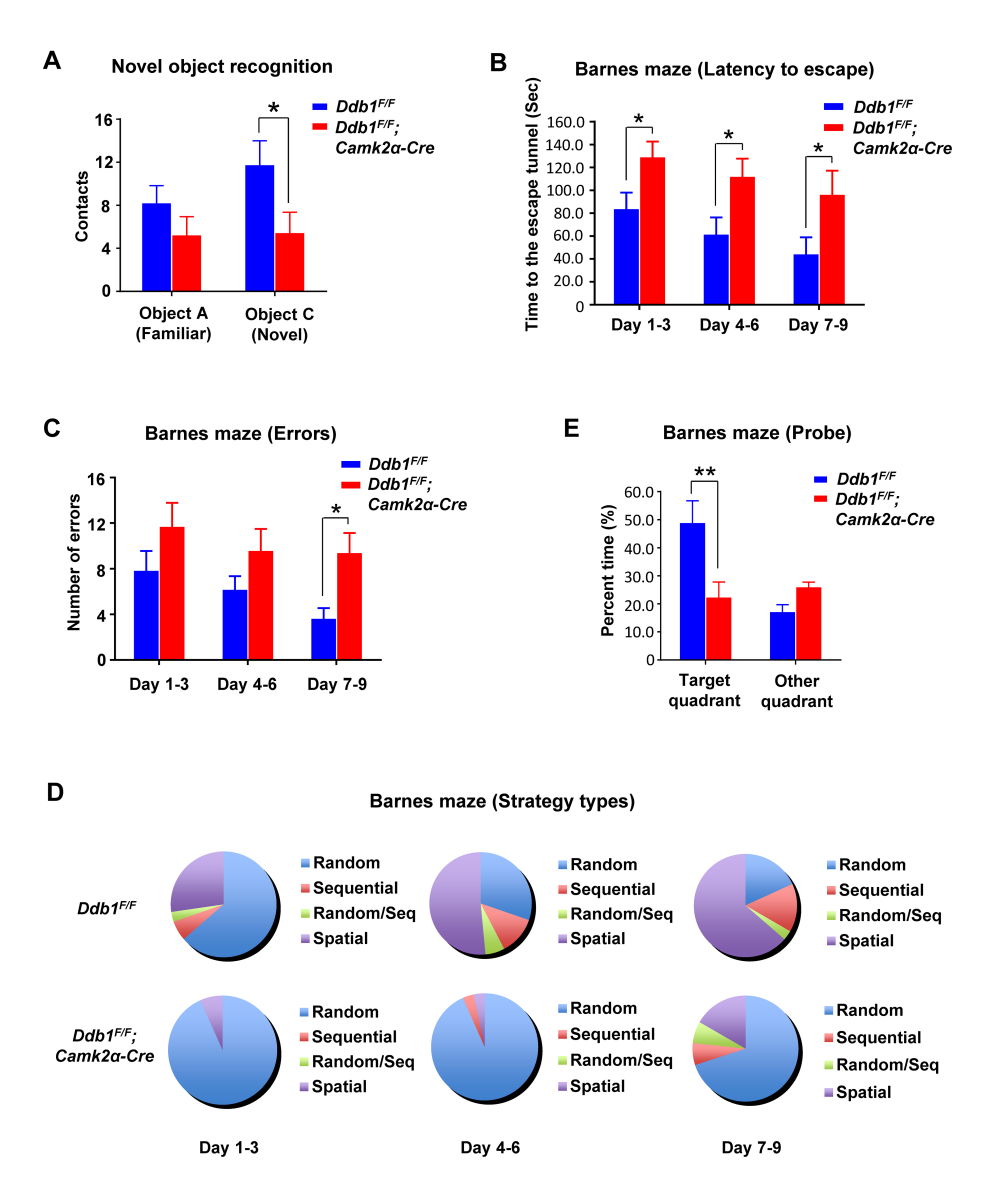
DDB1 deletion in mouse brain causes learning and memory defects. All behavior analysis data are shown as mean ± SEM. (A) Novel object recognition to test texture-associated short-term memory in mice. Contacts of mice towards the familiar (A) or novel (C) object were recorded after training. *Ddb1*^*F/F*^*;Camk2a-Cre* mice could not differentiate object C from A while *Ddb1*^*F/F*^ mice could (*Ddb1*^*F/F*^ *n* = 11, *Ddb1*^*F/F*^*;Camk2a-Cre n* = 10, *P* = 0.026). (B) Barnes maze. The time for *Ddb1*^*F/F*^*; Camk2a-Cre* mice (*n* = 10) or *Ddb1*^*F/F*^ *mice* (*n* = 11) to enter the escape tunnel were recorded during the training days (*P* < 0.05 for each block). Two-way repeated-measures *ANOVA* (genotype versus day) revealed a genotype effect (*F*_*(1*,*19)*_ = 6.518, *P* = 0.0194) and day effect (*F*_*(2*,*38)*_ = 6.318, *P* = 0.0043) for both groups. (C) Barnes maze. The errors (numbers of entrance into wrong tunnel) made by mice were recorded during the training days (*P* = 0.014 for block day 7–9). Two-way repeated-measures *ANOVA* revealed a genotype effect (*F*_*(1*,*17)*_ = 13.68, *P* = 0.0018) for both groups. (D) Barnes maze. Incidence distribution of strategy for mice to search the escape tunnel were recorded during the training days. Two-way repeated-measures *ANOVA* revealed a significant differences in strategy use (*F*_*(3*,*16)*_ = 29.00, *P* < 0.0001) and a genotype × strategy effect (*F*_*(3*,*16)*_ = 13.16, *P* = 0.0001) for both groups. (E) Barnes maze. The time for mice spent in the target quadrant (where the removed escape tunnel was located) were recorded on the probe day (*P* = 0.0027).

To test texture-associated short-term memory, we performed a novel object recognition task and found that *Ddb1*^*F/F*^*;Camk2α-Cre* mice had difficulty in differentiating a novel object from a familiar one compared to WT (Fig 1A). Additionally, in a cued and contextual fear conditioning task, which assesses the ability to learn and remember an association between environmental cues and aversive experiences, *Ddb1*^*F/F*^*;Camk2α-Cre* mice also exhibited significant deficits (S2A Fig).

To investigate spatial learning and memory, we performed the Barnes maze and Morris water maze tests on these animals. In the Barnes maze test, *Ddb1*^*F/F*^*;Camk2α-Cre* mice spent significantly more time and made more errors than WT during the 9-day training period (Fig 1B and 1C). WT mice were more likely to use a spatial-reference search strategy than either a sequential or random search strategy during the training process, but *Ddb1*^*F/F*^*;Camk2α-Cre* mice were inclined to use a random search strategy (Fig 1D). On the 10^th^ day (probe test), the escape tunnel was removed and the maze was divided into 4 quadrants. *Ddb1*^*F/F*^*;Camk2α-Cre* mice spent significantly less time in the target quadrant, where the escape tunnel was located, than WT mice did (Fig 1E). These data indicate that *Ddb1*^*F/F*^*;Camk2α-Cre* mice exhibited defects in both reference and working memory.

**Fig 2:**
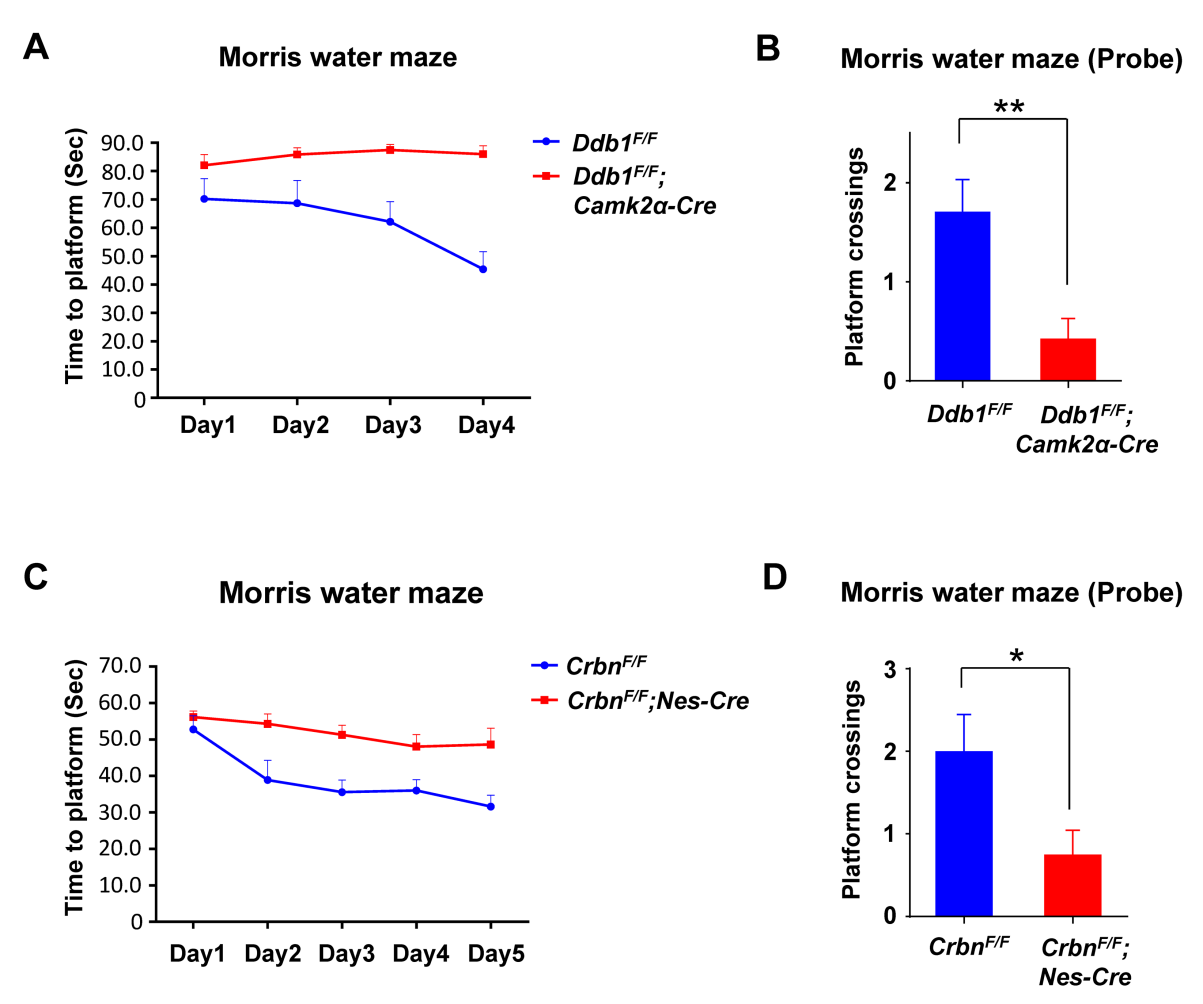
Similar learning and memory defects in DDB1 and CRBN deficient mice. All behavior analysis data are shown as mean ± SEM. (A) Morris water maze. The time for *Ddb1*^*F/F*^*;Camk2a-Cre* mice (*n* = 7) or *Ddb1*^*F/F*^ mice (*n* = 8) to find the hidden platform were recorded throughout all 4 training days. Two-way repeated-measures *ANOVA* revealed a major genotype effect (*F*_*(1*,*13)*_ = 29.60, *P* = 0.0001) for both groups. (B) Morris water maze. On the probe day, when platform was removed, there was a significant difference in the platform location crossings between the genotypes (*Ddb1*^*F/F*^ *n* = 8, *Ddb1*^*F/F*^*;Camk2a-Cre n* = 7, *P* = 0.0061). (C) Morris water maze. The time for *Crbn*^*F/F*^*;Nestin-Cre* mice (n = 9) or *Crbn*^*F/F*^ mice (*n* = 5) to find the hidden platform were recorded throughout all 5 training days. Two-way repeated-measures *ANOVA* revealed a huge genotype effect (*F*_*(1*,*12)*_ = 9.690, *P* = 0.009) and day effect (*F*_*(4*,*48)*_ = 11.63, *P* < 0.0001) for both groups. (D) Morris water maze. On the probe day, when the platform was removed, there was a significant difference in the platform location crossings between the genotypes (*Crbn*^*F/F*^ *n* = 5, *Crbn*^*F/F*^*;Nestin-Cre n* = 9, *P* = 0.0498).

In the Morris water maze test, mice were released from the edge of a water pool to locate a submerged hidden platform during a 4-day training period. *Ddb1*^*F/F*^*;Camk2α-Cre* mice took longer time to arrive at the platform in all trials. When analyzing their performance over the course of the experiment, *Ddb1*^*F/F*^*;Camk2α-Cre* mice showed no significant improvement, indicating their impaired spatial learning (Fig 2A). To evaluate the spatial memory, a probe trial was administered on the 5^th^ day. With the original platform removed, *Ddb1*^*F/F*^*;Camk2α-Cre* mice crossed the platform location much less frequently than WT mice, indicating their defective spatial memory (Fig 2B). Put together, these behavioral tests demonstrate that DDB1 deletion in mouse brain results in ID-like defects such as impaired learning and memory.

### CRBN deletion causes similar cognitive defects as well as DDB1 deletion

CRBN is a substrate receptor for CRL4 and its mutations are found in ID patients. It was reported previously that deletion of CRBN in the *Crbn*^*F/F*^*;Camk2α-Cre* or *Crbn*^*-/-*^ mice did not affect normal locomotor activity but caused a associative learning defect in a contextual fear-conditioning test [26]. We generated whole-brain CRBN KO mutant *Crbn*^*F/F*^*;Nestin-Cre* mice and verified this behavioral defect (S2B Fig), as well as the defect in DDB1 mutant mice (S2A Fig).

Like the DDB1 mutant mice, *Crbn*^*F/F*^*;Nestin-Cre* mice spent more time reaching the platform and showed less improvement over the trials during a 5-day training period in the Morris water maze test (Fig 2C). On the probe day, *Crbn*^*F/F*^*;Nestin-Cre* mice exhibited significantly fewer platform crossings (Fig 2D), suggesting that they were less able to remember the location of the platform compared to their WT littermates. We conclude that CRBN and DDB1 deficient mice displayed similar impairment in learning and memory, suggesting that this ID-like phenotype may be regulated by the CRL4^CRBN^ ubiquitin ligase.

### DDB1 deletion results in decreased CRBN protein level

**Fig 3:**
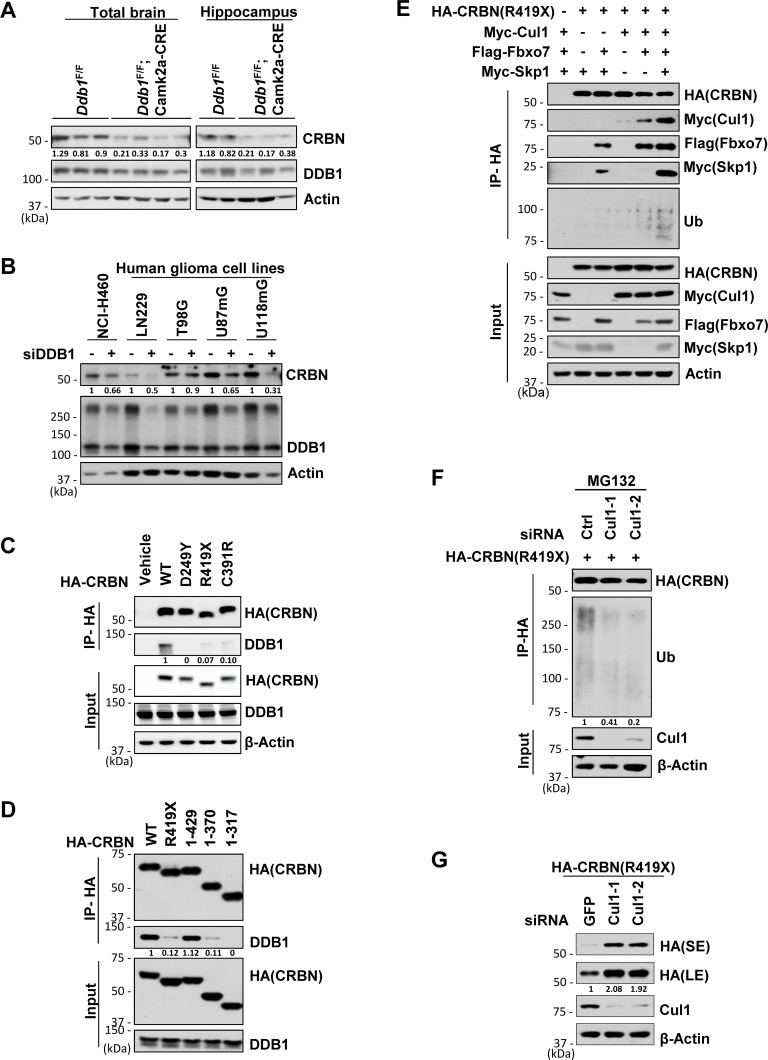
When dissociated from DDB1, CRBN and its ID-associated mutants are subjected to SCF^Fbxo7^-mediated destruction. Data are representative of experimental duplicates except for Fig 3A. (A) Western blot analysis for CRBN levels in protein extracts prepared from mouse brain (*Ddb1*^*F/F*^ n = 3; *Ddb1*^*F/F*^*;Camk2a-Cre n* = 4) or hippocampus (*Ddb1*^*F/F*^ n = 2; *Ddb1*^*F/F*^*;Camk2a-Cre n* = 3). Protein signals were measured by NIH Image J. Quantification of CRBN were standardized as the ratio of CRBN signals to the cognate actin signals, followed by normalization to the mean value of standardized CRBN signals in WT mouse brain or hippocampus. (B) Western blot analysis for CRBN levels in indicated cells transfected with siRNA for DDB1 or siRNA for GFP. siGFP, as a non-targeting control. Quantification of CRBN was normalized to the cognate actin signals. (C) Co-immunoprecipitation (co-IP) of HA-tagged WT or mutant CRBN with DDB1 in 293T cells. Whole-cell extracts from 293T cells over-expressing HA-CRBN constructs were immunoprecipitated with anti-HA affinity beads and analyzed by immunoblotting with indicated antibodies. Quantification of DDB1 pulled down by HA-CRBN in IP samples was normalized to the cognate DDB1 signals in input samples. (D) Identification of protein binding region of CRBN with DDB1 by immunoprecipitation of HA-tagged WT, R419X mutant or truncated CRBN with DDB1 after they were over-expressed in 293T cells. Quantification of DDB1 pulled down by HA-CRBN in IP samples was normalized to the cognate DDB1 signals in input samples. (E) Co-immunoprecipitation of CRBN^R419X^ with Fbxo7, Skp1, Cul1 and ubiquitin in 293T cells over-expressing HA-CRBN^R419X^, Flag-Fbxo7, Myc-Skp1 and Myc-Cul1. (F) Ubiquitination assay for CRBN^R419X^ by immunoprecipitation of CRBN^R419X^ with ubiquitin in 293T cells. 293T cells over-expressing HA- CRBN^R419X^ were transfected with siRNA for Cul1 or control and harvested for IP after treatment with MG132 (10 μM) for 6 hrs. Quantification of Ubiquitin pulled down by HA- CRBN^R419X^ was normalized to β-actin. (G) Western blot analysis for CRBN^R419X^ levels in 293T cells transfected with HA-CRBN^R419X^ along with siRNA for Ctrl or Cul1. L.E., long exposure. S.E., short exposure. Quantification of CRBN^R419X^ (L.E.) was normalized to β-actin.

To understand the functional crosstalk between DDB1 and CRBN, we examined the CRBN protein level in the brain or hippocampus of WT and *Ddb1*^*F/F*^;*Camk2α-Cre* mice. We observed that CRBN levels were reduced in the absence of DDB1 in mouse brain lysates (Fig 3A). To further confirm this result, we performed RNA interference (RNAi) in several cell lines, including human glioma cell lines LN229, U87mG and U118mG. Knockdown of DDB1 also resulted in reduced protein level of CRBN (Fig 3B). Therefore, DDB1 is required for the steady-state level of CRBN protein.

### ID-associated CRBN mutants fail to bind DDB1

A nonsense mutation in CRBN with a premature stop at amino acid 419, CRBN^R419X^, was identified in a large kindred to cause non-syndromic ID [23]. Recently, another missense variant in the CRBN, CRBN^C391R^, was identified in five individuals with severe ID from a consanguineous family [24]. By immunoprecipitaion assays, we found that both CRBN^R419X^ and CRBN^C391R^ associated very weakly with DDB1 (Fig 3C). Likewise, CRBN^D249Y^, a mutation located in close proximity to the DDB1 binding domain [28,29] and found in the lenalidomide-resistant ANBL-6 MM cell line [30], completely abolished its binding to DDB1 (Fig 3C). A further truncation analysis revealed that CRBN truncations (residues 1–317 and 1–370) exhibited a similar DDB1-binding defect to CRBN^R419X^, while a different truncation (residues 1–429) showed binding to DDB1 as strong as the full length protein (Fig 3D). This result suggests that loss of a 10 amino segment (419–429) in the DDB1 C-terminus might adversely affect the CRBN domain structure critical for DDB1 interaction, as would be predicted by the structure of the human CRBN-DDB1-lenalidomide complex [29] (S3A Fig).

### DDB1-binding-defective CRBN is targeted by SCF^Fbxo7^ for degradation

To investigate the mechanism underlying the decreased CRBN level caused by DDB1 deletion, we performed protein stability assays using the DDB1-binding-defective CRBN mutants. Compared with the WT protein, these mutant CRBN protein levels were decreased but can be restored by treating the cells with the proteasome inhibitor MG132 (S3B Fig). The cycloheximide (CHX) chasing assays confirmed a reduction of the protein stability of these CRBN mutants (S3C and S3D Fig). These results suggest that the mutant proteins are targeted by proteasomal degradation. The reduced stability of CRBN^R419X^ was also previously reported, but the CRL4 ligase was proposed to account for its turnover [31]. Since mutant CRBN fails to bind CRL4 and the protein level can also be restored by MLN4924, a neddylation inhibitor of all Cullin ubiquitin ligases (S3E Fig), we hypothesized that another Cullin family ubiquitin ligase other than CRL4 might target these mutants for degradation. Out of the seven human Cullins [32], Cul1 was found to interact with CRBN, though much more weakly compared to Cul4, when overexpressed in 293T cells (S3F Fig). The CRBN-Cul1 interaction was greatly enhanced when CRBN was abolished of its interaction with DDB1 or stabilized by MG132 (S3G Fig). Cul1 forms the SCF complex (also called CRL1) with Skp1 and Rbx1 to modify a large variety of substrates using various F-box adaptor protein [32,33]. It was reported that lenalidomide can increase CRBN stability [34] and also down-regulate the expression of a F-box protein Fbxo7, even to a greater extent than its down-regulation on IKZF1 and IKZF3 levels [35]. We found that Fbxo7 bound to the CRBN mutants (S3H Fig) and mediated the interaction of CRBN^R419X^ with Cul1 and Skp1 (Fig 3E). Overexpression of both Fbxo7 and Skp1 greatly enhanced the interaction between CRBN^R419X^ and Cul1, and the ubiquitination level of CRBN^R419X^ (Fig 3E; S3I and S3J Fig). Conversely, knockdown of Cul1 decreased the ubiquitination level of CRBN^R419X^ (Fig 3F), and increased the steady-state level of CRBN^R419X^ (Fig 3G). Taken together, we have identified a distinct E3 ligase that is not active on CRBN unless CRBN fails to bind DDB1. Considering DDB1 functions as a critical linker for CRL4 E3 ubiquitin ligase, these DDB1-binding defects in CRBN ID-associated mutations can disrupt their protein stability and the normal E3 function of CRL4^CRBN^ as well as DDB1 deletion. However, the question remains how DDB1 deletion or these CRBN mutations cause learning and memory defect.

### Inactivation of CRL4^CRBN^ destabilizes the BK channel

**Fig 4:**
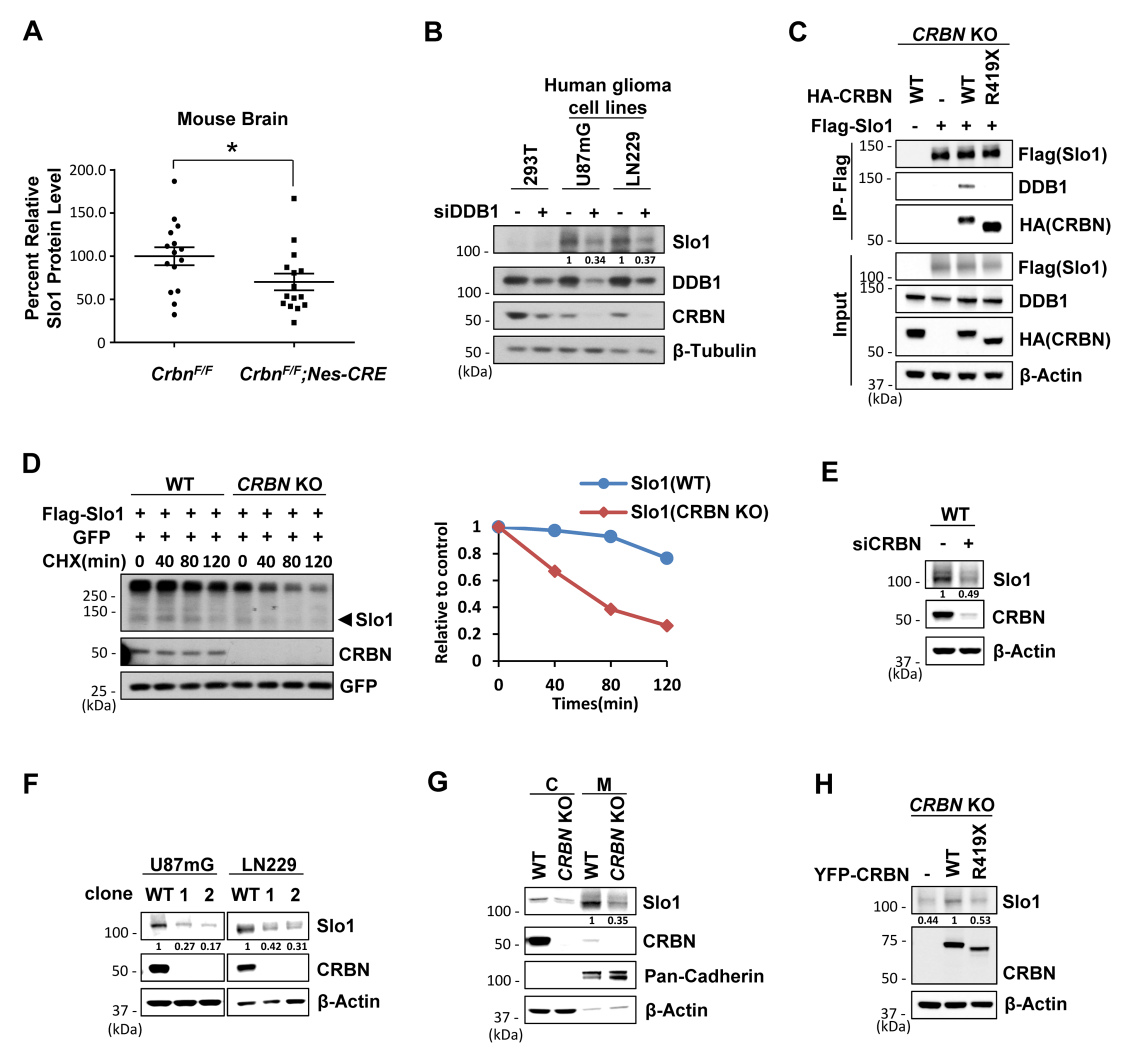
Deficiency of CRL4^CRBN^ promotes the destruction of BK channel α subunit. Data represent at least two independent experimental replicates. (A) Quantification of Slo1 level (detected by anti-Slo1, Novus) in brain extracts isolated from WT and *Crbn*^*F/F*^*;Nestin-Cre* mice (*n* = 15 for each group). Protein levels were standardized as the ratio of Slo1 signals to the cognate β-actin signals, followed by normalization to the mean value of standardized Slo1 signals in WT mice. Data are shown as mean ± SEM. *P* < 0.05. (B) Western blot analysis for endogenous Slo1 levels in indicated cells transfected with siRNA for Ctrl or DDB1. Quantification of Slo1 was normalized to β-tubulin. (C) Co-immunoprecipitation of Slo1 with WT or mutant CRBN and DDB1 in *CRBN* KO 293T cells. Cells were transfected with expression vectors encoding indicated genes followed by IP with Flag affinity beads. (D) CHX (100 μg/ml) chasing assays for the stability of Slo1 in WT or *CRBN* KO 293T cells. Cells were transfected with equivalent Flag-Slo1 along with equivalent GFP and harvested at the indicated time following CHX (Left).Arrowhead indicates the band of Slo1 used for quantification. Plot shows the quantification of Slo1 normalized to GFP signals and relative to the normalized Slo1 signals at the 0 min time point (Right). (E) Western blot analysis for Slo1 levels in LN229 glioma cells transfected with siRNA for ctrl or CRBN. Quantification of Slo1 was normalized to β-actin. (F) Western blot analysis for Slo1 levels in U87mG and LN229 glioma cells after CRISPR/Cas9-mediated *CRBN* gene editing. Quantification of Slo1 was normalized to β-actin. (G) Western blot analysis for Slo1 levels in cytoplasm or membrane extracts prepared from WT or *CRBN* KO U87mG cells. Pan-Cadherin, as a marker of membrane proteins. Quantification of membrane Slo1 was normalized to Pan-Cadnerin. (H) Western blot analysis for Slo1 levels in *CRBN* KO U87mG cells stably expressing vehicle and WT or mutant CRBN. Quantification of Slo1 was normalized to β-actin.

Loss of the BK channel α subunit (Slo1) was reported to cause impairment in spatial cognitive function [25]. Considering Slo1 is a direct substrate of CRL4^CRBN^, we examined the Slo1 level in DDB1 or CRBN deficient mouse brain lysates. Slo1 protein abundance was statistically reduced in mouse brain with either DDB1 or CRBN depleted (Fig 4A; S4A and S4B Fig). Consistent with the animal study, knockdown of DDB1 or Cul4A/4B expression in 293T cells dramatically reduced the abundance of exogenously expressed Slo1 protein (S4C Fig). To further confirm the reduction by endogenous Slo1, we checked the expression of endogenous Slo1 in a series of cell lines, using glioma cells transfected with sgRNA for Slo1 as a negative control. As expected, Slo1 is not expressed in 293T and MM1S cells, but highly expressed in glioma cells such as U87mG and LN229 (S4D Fig). Knockdown of DDB1 led to reduced endogenous CRBN and Slo1 protein abundance in both U87mG and LN229 cells (Fig 4B). Therefore, like CRBN, the steady-state level of Slo1 is also maintained by an intact CRL4 complex in cells and mouse brain.

To determine the role of CRBN and its ID-associated mutations in Slo1 regulation, we generated *CRBN* KO 293T cells using the CRISPR-Cas9 gene editing tool (S4E Fig). Deletion of CRBN resulted in the dissociation of Slo1 from binding to CRL4 (Fig 4C) and a profound decrease of the half-life of the exogenously expressed Slo1 (Fig 4D). Re-introducing WT CRBN but not mutant CRBN^R419X^ in these KO cells restored both the binding to CRL4 and the half-life of Slo1 (Fig 4C; S4F Fig). Endogenous Slo1 level was also decreased after CRBN was knocked down by RNAi or knocked out by CRISPR-Cas9 gene editing in glioma cells (Fig 4E and 4F; S4G Fig). As BK channel functions as a neuronal excitability regulator on the cell membrane, we extracted total membrane proteins and verified that the membrane Slo1 levels were also decreased in *CRBN* KO U87mG cells (Fig 4G) and LN229 cells (S4H Fig). Stable expression of WT CRBN but not mutant CRBN^R419X^ in the *CRBN* KO cells could obviously restore the Slo1 levels. (Fig 4H). Put together, we conclude that CRBN and its interaction with CRL4 are essential for the steady-state level of Slo1.

### CRBN mutations redirect BK channel to SCF^Fbxo7^ for proteasomal degradation

**Fig 5:**
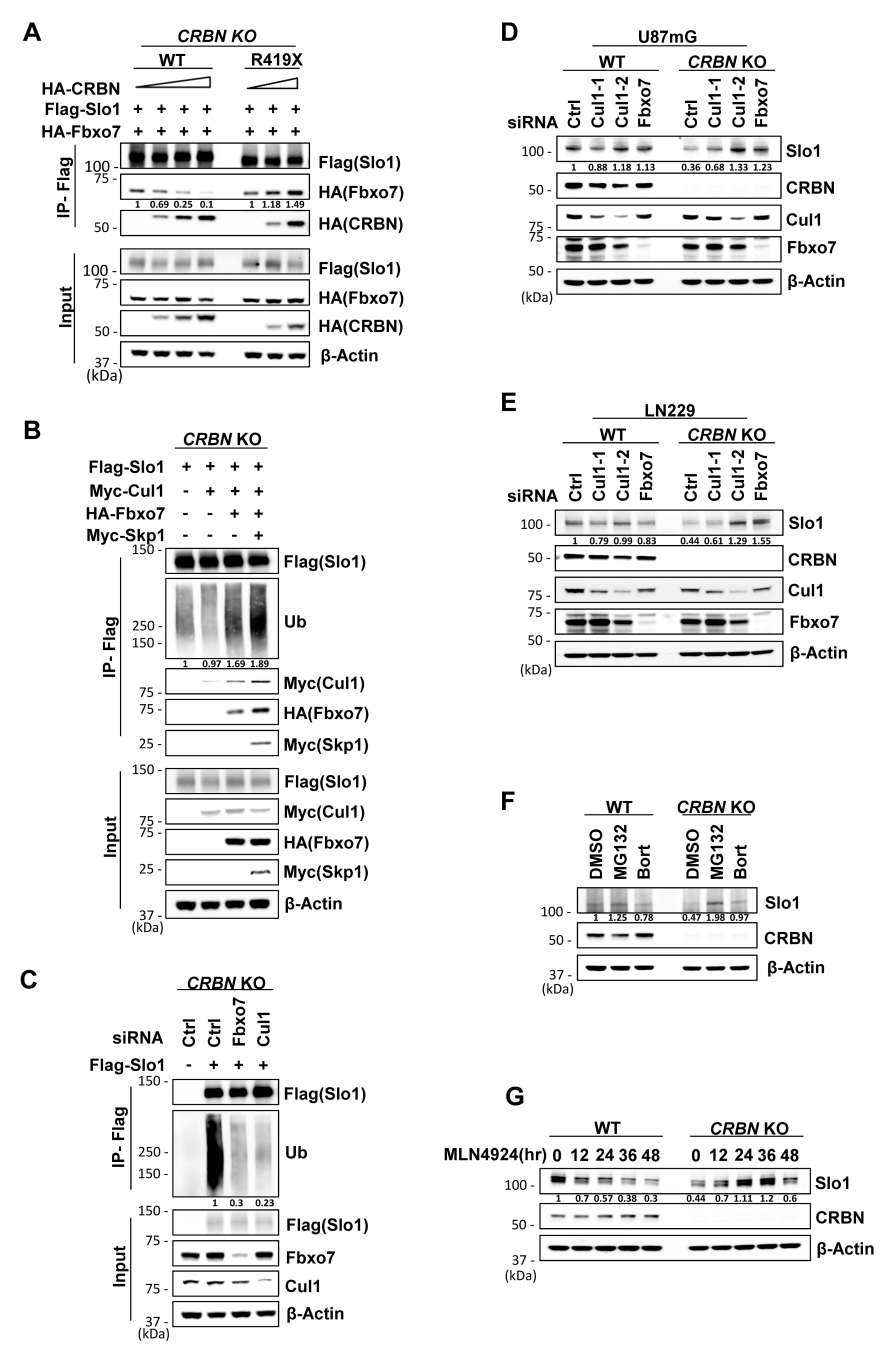
When unbound to CRL4, BK channel α subunit is redirected to SCF^Fbxo7^ for proteasomal degradation. Data represent at least two independent experimental replicates. (A) Co-immunoprecipitation of Slo1 with Fbxo7 and CRBN in *CRBN* KO 293T cells. Cells were transfected with Flag-Slo1, HA-Fbxo7 and increasing amounts of HA tagged WT or mutant CRBN and harvested for IP with Flag affinity beads. Quantification of Fbxo7 pulled down by Flag-Slo1 in IP samples was normalized to the cognate Fbxo7 signals in input samples. (B) Co-immunoprecipitation of Slo1 with SCF^Fbxo7^ complex and ubiquitin in *CRBN* KO 293T cells. Cells were transfected with expressing vectors encoding indicated genes and harvested after treatment with MG132 (10 μM) for 6 hr. Quantification of endogenous ubiquitin pulled down by Flag-Slo1 was normalized to β-actin. (C) Ubiquitination assay for Slo1 by co-immunoprecipitation of Slo1 with ubiquitin in *CRBN* KO 293T cells. Cells were transfected with siRNA for Ctrl, Fbxo7 or Cul1 and harvested after treatment with MG132 (10 μM) for 6 hrs. Quantification of endogenous ubiquitin pulled down by Flag-Slo1 was normalized to β-actin. (D) Western blot analysis for Slo1 levels in WT or *CRBN* KO U87mG cells transfected with siRNA for Ctrl, Fbxo7 or Cul1. Quantification of Slo1 was normalized to β-actin. (E) Western blot analysis for Slo1 levels in WT or *CRBN* KO LN229 cells transfected with siRNA for Ctrl, Fbxo7 or Cul1. Quantification of Slo1 was normalized to β-actin. (F) Western blot analysis for Slo1 levels in WT or *CRBN* KO LN229 cells treated with MG132 (20 μM) for 8 hrs or Bortezomib (5 nM) for 24 hrs. DMSO, as vehicle control. Quantification of Slo1 was normalized to β-actin. (G) Western blot analysis for Slo1 levels in WT or *CRBN* KO LN229 cells treated with MLN4924 (0.2 μM) for indicated time. Quantification of Slo1 was normalized to β-actin.

Considering CRBN deletion has little effect on Slo1 mRNA level in both U87mG and LN229 cells (S5A Fig), the decreased Slo1 level is achieved via a posttranslational mechanism. We then tested whether Slo1 was redirected to SCF^Fbxo7^ for turnover along with CRBN^R419X^, when unbound to CRL4. Immunoprecipitation assays revealed that Slo1 interacted with Fbxo7 strongly when CRBN was deleted or mutated (Fig 5A; S5B Fig). WT CRBN instead diminished the interaction of Slo1 and Fbxo7 (Fig 5A) and of Slo1 and Cul1 (S5C Fig). We further found that Fbxo7 bound to the RCK1 and RCK2 domains of Slo1 (S5D Fig), the same domains interacting with CRBN [18], which is consistent with the competitive targeting of Slo1 by Fbxo7 and WT CRBN (Fig 5A).

In the ubiquitination assay, Slo1 formed a complex with SCF^Fbxo7^ and was still ubiquitinated in *CRBN* KO 293T cells (Fig 5B). The ubiquitination level of Slo1 increased along with overexpression of SCF^Fbxo7^ components (Fig 5B), and decreased after knockdown of Fbxo7 or Cul1 (Fig 5C). Importantly, reduced Slo1 protein level in *CRBN* KO U87mG and LN229 cells could be restored by knockdown of Fbxo7 or Cul1 (Fig 5D and 5E). Likewise, treatment with proteasome or neddylation inhibitors (MG132, bortezomib [36] or MLN4924 [37]) in *CRBN* KO glioma cells could also increase the Slo1 levels, though treatment with bortezomib or MLN4924 in WT cells for excessive time led to decreased Slo1 levels, likely due to compound cytotoxicity (Fig 5F and 5G). Put together, these results demonstrate that Slo1, upon dissociated from CRL4 by CRBN mutations, is redirected to SCF^Fbxo7^ for proteasomal destruction.

### CRBN mutations lead to density-dependent decrease of BK channel currents

**Fig 6:**
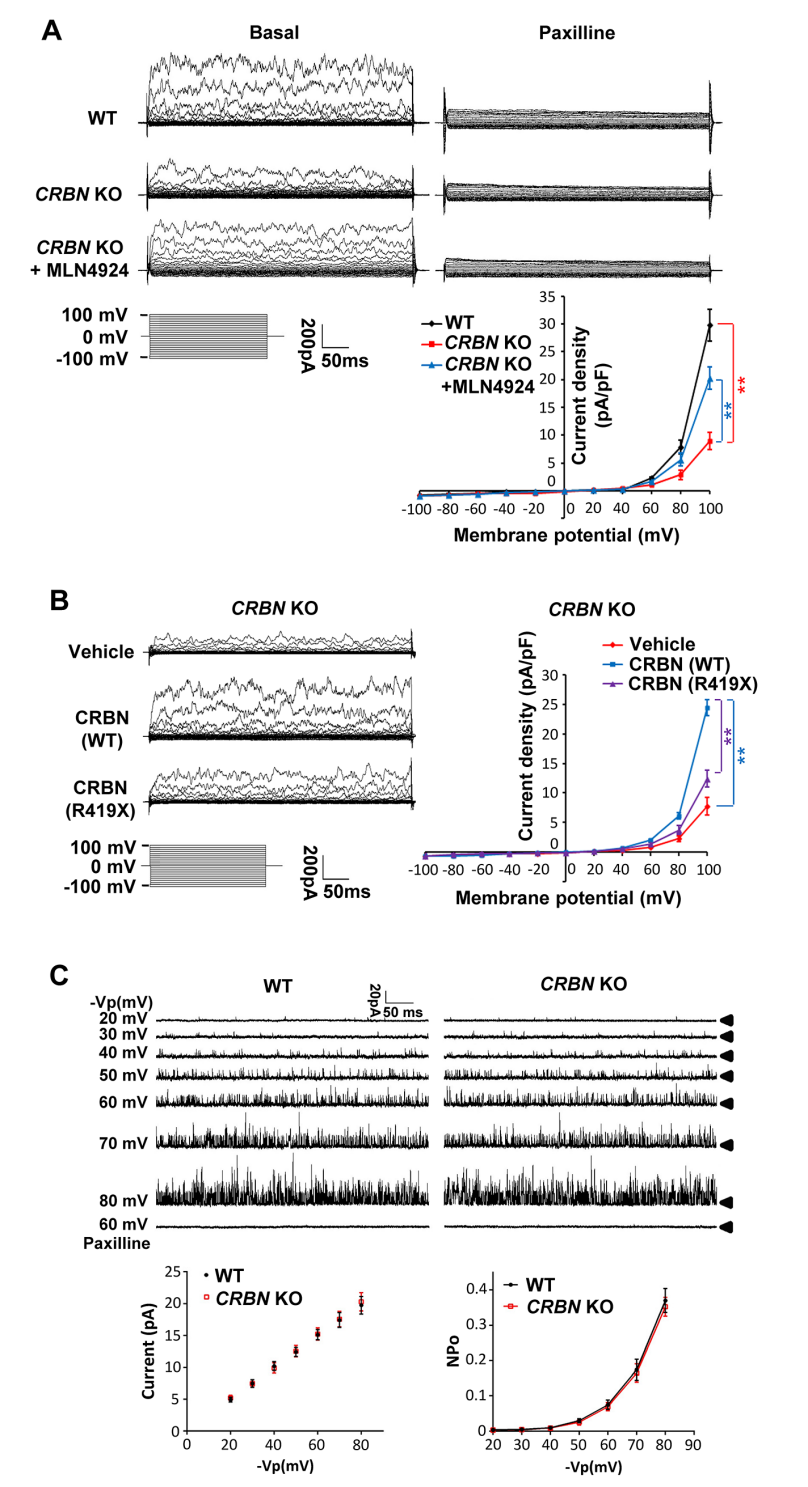
Currents of BK channel are decreased in CRL4^CRBN^ deficient glioma cells. (A) The whole cell currents of U87mG cells were detected by voltage clamp. BK currents were presented as the difference between the currents under basal condition and after treatment of paxilline (10 μM, BK channel blocker) for at least 10 mins. The I-V curve of BK currents in WT cells, *CRBN* KO cells and *CRBN* KO cells treated with MLN4924 (0.1 μM) for 24 hrs were shown on the bottom right (*n* = 5 for each group, *P* < 0.01). (B) The whole cell currents in *CRBN* KO cell lines stably expressing vehicle, WT CRBN and R419X mutation-bearing CRBN were detected. The I-V curve of BK currents (paxalline-subtracted currents) in these cell lines were shown on the right panel (*n* = 5 for each group, *P* < 0.01). (C) BK single-channel currents were recorded with a pipette voltage from 20 mV to 80mV using the excised inside-out configuration and were then identified by its pharmacological sensitivity. Mean single channel current and mean NPo versus voltage relationships were derived from data sets exemplified in the current traces (*n* = 5 for each group).

To explore the physiological significance of the Slo1 turnover by CRBN mutations, the whole cell currents of *CRBN* KO U87mG and LN229 cells were measured by voltage clamp. BK currents were presented as the difference between the currents under baseline condition and after treatment with paxilline, a BK channel blocker [38,39] (Fig 6A; S6A Fig). The BK currents were significantly decreased in CRBN-depleted cells compared to isogenic WT cells, and the difference was largely offset by treating the mutant cells with MLN4924 (Fig 6A), likely as a result from an increased expression of Slo1 (Fig 5G). However, MLN4924 treatment did not affect BK currents in the WT cells (S6B Fig). Additionally, the decreased whole cell currents in *CRBN* KO cells could also be significantly enhanced by stable expression of WT CRBN, but not as much by expression of the mutant CRBN^R419X^ (Fig 6B). To rule out individual BK channel activity defect, BK single-channel currents were recorded using the excised inside-out configuration. No significant difference of single channel conductance and open probability was found between WT and *CRBN* KO cells (Fig 6C). These results suggest that CRBN mutations decrease BK channel activity on cell membrane by destabilizing the protein.

### Impaired learning and memory in CRBN mutant mice is rescued by BK activation

**Fig 7:**
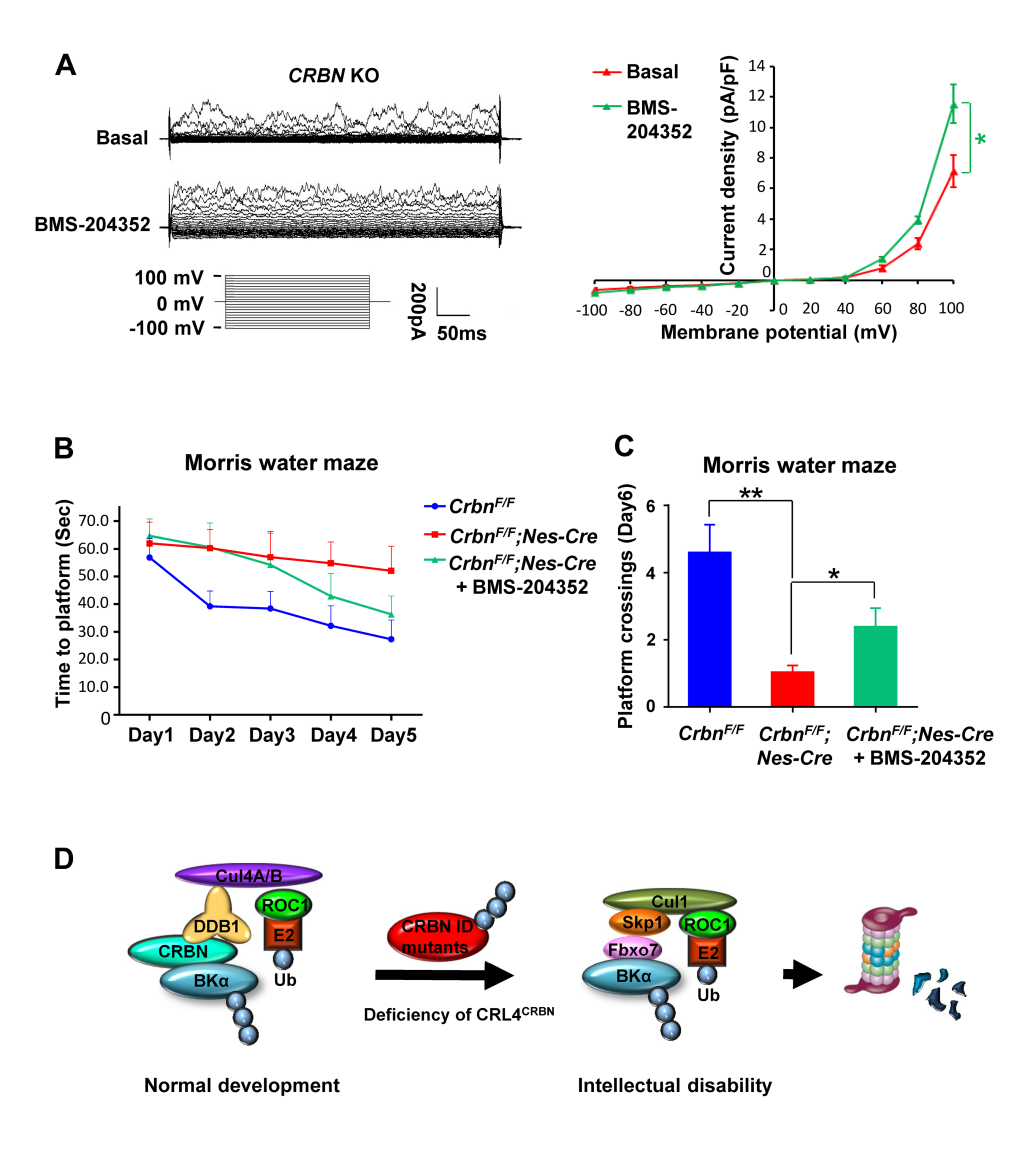
Impaired spatial memory and learning in CRL4^CRBN^ deficient mice is partially rescued by a BK channel opener. (A) The whole cell currents in *CRBN* KO U87mG cells were detected. The I-V curve of BK currents (paxalline-subtracted currents) before and after BMS-204352 (10 μM, BK channel opener) treatment were shown on the right panel (*n* = 5 for each group, *P* < 0.05). (B) The time for *Crbn*^*F/F*^*;Nestin-Cre* mice (*n* = 7) or *Crbn*^*F/F*^ mice (*n* = 8) to find the hidden platform throughout all 5 training days of the Morris water maze. Two-way repeated-measures *ANOVA* revealed a significant genotype effect (*F*_*(1*,*13)*_ = 4.906, *P* = 0.045) and day effect (*F*_*(4*,*52)*_ = 4.750, *P* = 0.0024) for both groups. BMS-204352 treatment to *Crbn*^*F/F*^*;Nestin-Cre* (*n* = 7) mice narrowed the gap between the genotypes (*F*_*(1*,*13)*_ = 2.649, *P* = 0.128). (C) On the probe day of the Morris water maze, when the platform was removed, there was a significant difference in the platform location crossings between the genotypes (*Crbn*^*F/F*^ *n* = 8, *Crbn*^*F/F*^*;Nestin-Cre n* = 7, *P* = 0.0027). The phenotype was partially corrected by BMS-204352 treatment that increased memory in *Crbn*^*F/F*^*;Nestin-Cre* mice (*Crbn*^*F/F*^*;Nestin-Cre n* = 7, *Crbn*^*F/F*^*;Nestin-Cre* + BMS-204352 *n* = 7, *P* = 0.043). (D) A schematic model for ID caused by deficiency of CRL4^CRBN^.

To demonstrate the reduced BK activity accounts for the impaired learning and memory of the CRBN mutant mice, we performed the Morris water maze tests on *Crbn*^*F/F*^*;Nestin-Cre* mice treated with a BK channel opener, BMS-204352 [20,40,41]. This compound was validated to be able to activate the residual membrane BK channels to elevate the currents in *CRBN* KO cells (Fig 7A). In a bigger platform and with a longer training period for better quantitative comparison, BMS-204352-treated *Crbn* mutant mice spent significantly less time than vehicle-treated mutant mice to reach the platform, especially in the last 2 training days (Fig 7B) and exhibited more frequent platform crossings on the probe day (Fig 7C). Unfortunately, restoring Slo1 protein levels in the CRBN mutant mouse brain with bortezomib or MLN4924 cannot be achieved, due to these two compounds’ toxicity and failure to pass blood-brain barrier.

## Discussion

### Dual ubiquitination of BK channel under physiological and pathological conditions

In a physiological context, BK channel is recruited by CRBN to the CRL4 ubiquitin ligase for ubiquitination and retention in the ER compartment [18]. When this post-translational modification is disrupted in ID patients with CRBN mutations, BK channel is rerouted to an unrelated SCF^Fbxo7^ ubiquitin ligase for ubiquitination and, instead of ER retention, proteasomal degradation (Fig 7D). The differential targeting under normal and pathological conditions would ensure the limited distribution of the BK channel in the cell surface and thus the exquisite control of its activities and neuronal excitability. The dual ubiquitination of the same substrate is not an unusual mechanism for regulation of protein stability. For example, Myc oncoprotein can be ubiquitinated by the SCF^β-TrCP^ ubiquitin ligase to prevent its turnover by SCF^Fbw7^-mediated ubiquitination [42]. Cand1 can promote exchange of Fbw7 for β-TrCP in SCF complex [43], further modulating the cellular repertoire of SCF complex for control of Myc stability and cell proliferation. While many details on the differential targeting still need working out, it is clear that critical factors including BK channel and Myc are subjected to highly orchestrated regulation even just at the level of post-translational modification.

### SCF^Fbxo7^ targets BK channel pore subunit for degradation

BK channel is composed of four α subunits (Slo1), which form the ion channel pore, and auxiliary subunits, including four regulatory β (β1–4) subunits that are expressed in various tissues [19]. The β1 subunit, primarily distributed in smooth muscle cells, controls cell surface trafficking by distinct domains of N-terminus [44]. The β1 subunit is ubiquitinated for proteolysis in diabetes-like conditions [45], which may weaken its ability to dynamically assemble with Slo1 at the membrane [46]. However, ubiquitination of BKβ1 alone may not necessarily cause a reduced level of Slo1, especially in brain cells [47]. Here we report for the first time that the protein level of the pore-forming Slo1 was directly regulated by the ubiquitin-proteasome system, and that this post-translational destruction of Slo1 decreases BK current. It will be interesting to examine the effects and conditions of SCF^Fbxo7^-mediated ubiquitination and turnover of Slo1 in the alteration of the action potential (AP) shape and duration in neurons [47,48] as well as the Ca^2+^-dependent modulation of neurotransmitter release at presynaptic terminals [49].

### CRL4^CRBN^ mutations cause ID by altering BK channel activity

ID is a clinically diverse disorder with heterogeneous genetic inheritance. We first prove that DDB1 is a critical regulator in learning and memory using mouse model. DDB1 and CRBN deletion in mouse brain can cause similar ID-like phenotypes. Then we provide mechanistic insight into ID pathogenesis in patients with CRBN mutations by demonstrating that they are DDB1-binding defective, which makes them unstable due to SCF^Fbxo7^-mediated degradation. At last, we identify that the protein levels and activities of the BK channel are dysregulated by mutant CRBN. Moreover, a BK channel opener can significantly improve the learning and memory impairment in CRBN mutant mice. Therefore, BK channel may be one of the several substrates of CRL4^CRBN^ that are disrupted by changes in protein ubiquitination [50–52], due to CRBN or Cul4B ID mutations. However, whether enhancing BK level in brain is efficient and sufficient to rescue ID-like phenotypes in CRL4^CRBN^ deficient mice, still remains to be further elucidated. Whether low BK channel activity is found in ID patients, particularly those with CRBN or Cul4B mutations, still needs in-depth clinical investigation. Since there is currently no effective treatment for ID, targeting BK channel activity might provide a potential therapeutic intervention for this neurological disorder.

## Materials and methods

### Ethics statement

Animal experiments were performed in Zhejiang University and the Scripps Institute with approval from Institutional Animal Care and Use Committee from both institutions (approval number 15614 for Zhejiang University) and comply with the Guide for the Care and Use of Laboratory Animals (NIH publication no.86-23, revised 1985).

### Compounds and antibodies

MG132 (S2619, Selleck Chemicals), bortezomib (S1013, Selleck Chemicals), MLN4924 (M2189, Abmol), Cycloheximide (R750107, Sigma-Aldrich), Paxilline (P2928, Sigma-Aldrich) and BMS-204352 (SML1313, Sigma-Aldrich) were dissolved in DMSO. Anti-CRBN (SAB045910, Sigma-Aldrich), anti-DDB1 (37–6200, Invitrogen), anti-actin (1844–1, Epitomics), anti-β-actin (4967, cell Signaling Technology; 5779–1, Epitomics), anti-β-tubulin (32–2600, Invitrogen), anti-Pan-Cadherin (4068, Cell Signaling Technology), anti-ubiquitin (sc-271289, Santa Cruz), anti-Cul1 (AP16324b, Abgent), anti-Fbxo7 (ab84129, Abcam), anti-Cul4A (A300-739A, Bethyl Laboratories), anti-Cul4B (2527–1,Epitomics), anti-Slo1 (NBP1-48250, Novus Biologicals), anti-mSlo1 (Millipore, MABN70), anti-HA (H6908; H3663, Sigma-Aldrich), anti-Flag (F3165, Sigma-Aldrich), anti-Myc (2272, Cell Signaling Technology), anti-GFP (sc-8334, Santa Cruz), anti-HA agarose (E6779, Sigma-Aldrich), anti-Flag M2 agarose (A2220, Sigma-Aldrich), goat anti-mouse IgG-HRP (sc-2005; Santa Cruz) and goat anti-rabbit IgG-HRP (sc-2004; Santa Cruz) were used following the manufacturers’ protocol.

### Animals

Animals were bred in a pathogen-free and temperature-controlled barrier facility with a 12-h-light/dark cycle and free access to food and water. All mice were bred on a mixed 129×C57BL/6J background. Generation and characterization of *Ddb1*^*F/F*^ and *Ddb1*^*F/F*^*;Camk2α-Cre* mice have been described previously[18,27]. *Crbn*^*F/F*^ mice [26] were purchased from The Jackson Laboratory (Bar Harbor, ME) and crossed with *Nestin-Cre* strains. The efficiency and specificity of CRBN deletion in the brain was confirmed by PCR (forward primer: 5’-CAG TCA GAT GGG TAA GGA GCA-3’; reverse primer: 5’-AAG CAG CTC CGT AAT GCT G-3’) and further confirmed by Western blotting. The homozygous KO mouse would generate a PCR product of 467 bp while the heterozygous and WT mouse had a 387 bp PCR product.

### Locomotor activity test

*Ddb1*^*F/F*^ (*n* = 16, 10 males and 6 females, 5–8 month) and *Ddb1*^*F/F*^*;Camk2α-Cre* (*n* = 15, 8 males and 7 females, 5–8 month) mice were tested for locomotor activity in polycarbonate cages (42×22×20 cm) placed into frames (25.5×47 cm) mounted with two levels of photocell beams at 2 and 7 cm above the bottom of the cage (San Diego Instruments, San Diego, CA). These two sets of beams allowed for the recording of both horizontal (locomotion) and vertical (rearing) behaviors. A thin layer of bedding material was applied to the bottom of the cage. Each mouse was tested for 60 minutes to evaluate its locomotor activity.

### Light/Dark transfer test

*Ddb1*^*F/F*^ (*n* = 16, 10 males and 6 females, 5–8 month) and *Ddb1*^*F/F*^*;Camk2α-Cre* (*n* = 15, 8 males and 7 females, 5–8 month) mice were subjected to the light/dark transfer procedure to assess anxiety-like behaviors by capitalizing on the conflict between exploration of a novel environment and the avoidance of a light open field. The apparatus was a rectangular box made of Plexiglas divided by a partition into two environments. One compartment (14.5×27×26.5 cm) was dark (8–16 lux) and the other compartment (28.5×27×26.5 cm) was highly illuminated (400–600 lux) by a 60 W light source located above it. The compartments were connected by an opening (7.5×7.5 cm) located at floor level in the center of the partition. Mice were placed in the dark compartment to start the five minutes test. The time spent in the light compartment was recorded. Duration in the light compartment was used as indicator of anxiety-like behavior.

### Vision testing

The visual cliff test provided a measure of visual acuity by evaluating the ability of *Ddb1*^*F/+*^*;Camk2α-Cre* (*n* = 5, 3 males and 2 females, 5 month) and *Ddb1*^*F/F*^*;Camk2α-Cre* (*n* = 5, 3 males and 2 females, 5 month) mice to see a drop-off at the edge of a horizontal surface. In this apparatus, there was the visual appearance of a cliff but in fact the Plexiglas provided a solid horizontal surface. If the animal saw the cliff, it would step down onto the “safe” side (the horizontal checkered surface) in most trials. A blind animal would just as often step down onto the “negative” side (the vertical appearing surface), i.e. make 50% correct and 50% incorrect choices. Each mouse was placed onto the center ridge, and the side onto which the animal stepped down was recorded. Six consecutive trials were used for each mouse and the percent of correct choices was calculated for each mouse.

### Novel object recognition test

*Ddb1*^*F/F*^ (*n* = 11, 7 males and 4 females, 6–9 month) and *Ddb1*^*F/F*^*;Camk2α-Cre* (*n* = 10, 5 of either sex, 6–9 month) mice were individually habituated to a 51×51×39 cm open field for 5 minutes and then tested with two identical objects (A and B) placed in the field (two clear plastic cylinders 6×6×16 cm half filled with glass marbles). Individual animal was allowed to explore for 5 minutes in the present of objects. After two such trials (each separated by 1 minute in a holding cage), the mouse was tested by object novelty recognition test in which a novel object (C) replaced one of the familiar objects (B). All objects and the area were thoroughly cleaned with 70% ethanol between trials to remove odors. Behavior was video recorded and scored for contacts (touching with nose or nose pointing at object within 4 cm of object).

### Cued and contextual fear conditioning

*Ddb1*^*F/F*^ (*n* = 15, 8 males and 7 females, 5–8 month) and *Ddb1*^*F/F*^*;Camk2α-Cre* (*n* = 16, 7 males and 9 females, 5–8 month) or *Crbn*^*F/F*^ (*n* = 9, female, 5–6 month) and *Crbn*^*F/F*^*;Nestin-Cre* (*n* = 9, female, 4–7 month) mice were trained to associate a novel environment (context) and a previously neutral stimulus (conditioned stimulus, a tone) with an aversive foot shock stimulus. Testing was performed in the absence of aversive stimulus. Conditioned animals, when exposed to the conditioned stimuli, were tended to refrain from all but respiratory movements by freezing. Freezing responses could be triggered by exposure to context in which the shock was received (context test) or conditioned stimulus (CS+ test). Conditioning took place in freeze monitor chambers housed in sound-proof boxes. The conditioning chambers (26×26×17 cm) were made of Plexiglas with speakers and lights mounted on two opposite walls. The chambers were installed with a shockable grid floor. On day 1, mice were placed in the conditioning chamber for five minutes in order to habituate them to the apparatus. On day 2, mice were exposed to the context and conditioned stimulus (30 seconds, 3000 Hz, 80 dB sound) in association with foot shock (0.70 mA, 2 second, scrambled current). Specifically, mouse received two shock exposures, both in the last 2 seconds of a 30 second tone exposure, during a 5.5-minute session. On day 3, contextual conditioning (as determined by freezing behavior) was measured in a 5-minute test in the chamber where the mice were trained (context test). On day 4, the mice were tested for cued conditioning (CS+ test). The mice were placed in a novel context for 3 minutes, after which they were exposed to the conditioned stimulus (tone) for 3 minutes. For this test, the chamber was disguised with new walls (black opaque plastic creating a triangular-shaped compartment in contrast to a clear plastic square compartment), a new floor (black opaque plastic in contrast to metal grid) and a novel odor (drop of orange extract under the floor). Freezing behavior, i.e. the absence of all voluntary movements except breathing, was measured in all sessions by a validated computer-controlled recording of photocell beam interruptions.

### Barnes maze

The Barnes maze apparatus is an opaque Plexiglas disc 75 cm in diameter elevated 58 cm above floor by a tripod. Twenty holes, 5 cm in diameter, were located 5 cm from the perimeter and a black Plexiglas escape box (19×8 ×7 cm) was placed under one of the holes. Distinct spatial cues were located all around the maze and kept constant throughout the study. A training session was performed by placing *Ddb1*^*F/F*^ (*n* = 11, 7 males and 4 females, 7–10 month) and *Ddb1*^*F/F*^*;Camk2α-Cre* (*n* = 10, 5 of either sex, 7–10 month) mice in the escape box for one minute in the first day. Testing session started after the habituation period. At the beginning of each session, the mouse was placed in the middle of the maze in a 10 cm high cylindrical black start chamber. After 10 seconds the start chamber was removed to free mouse exploring maze with a buzzer (80 dB) and a light (400 lux) turning on. The session ended when mouse entered the escape tunnel or after 3 min elapse. When mouse entered the escape tunnel, buzzer was turned off and mouse was allowed to remain in the dark for one minute. If the mouse failed to enter the tunnel by itself it was gently put in the escape box for one minute. The hole to place underneath tunnel was fixed for the same mouse but randomly determined. Mice were tested once a day in a 9 days’ acquisition portion with video typing. Latency to escape, the number of errors made and strategies chosen per session were measured from record. Errors are defined as nose pokes and head deflections over any hole that does not have tunnel. Strategies were determined by examining each mouse's daily session and classifying it as either “Random”—localized hole searches separated by crossings through the center of the maze (no systematic search pattern), “Sequential”—systematic hole searches (every hole or every other hole) in a clockwise or counterclockwise direction, “Random/Sequential”—systematic hole searches separated by crossings through the center of the maze, or “Spatial”—reaching the escape tunnel directly with both error and distance (number of holes between the first hole visited and the escape tunnel) scores of less than or equal to 3. For the 10th test (probe test), escape tunnel was removed and mouse was allowed to freely explore the maze for 3 minutes. The time spent in each quadrant was determined and the time spent in the target quadrant (the one originally containing the escape box) was compared with that in the other three quadrants.

### Morris water maze

Morris water maze was comprised of a circular tank (150 cm diameter, 50 cm height) with 34 cm deep warm milky water (22±1°C), a platform submerged 1 cm below the water surface, a video camera and a computer. During the training phase, mice were placed facing the wall of the pool and permitted to swim for 60 or 90 seconds to locate the platform. If the mice failed, they were placed in the platform for 10 seconds to learn the location. 3–4 trials with different starting positions per day were performed for each mouse with recording of time to locate the platform. After training, test was repeated in the pool where the platform was removed on the probe day. Test parameters were listed as follows (Table 1).

**Table 1:** Parameters of Morris water maze.

	Mice groups	Sex / Age	Platform diameter	Training		Probe	
				Trials /Day	Time /Trail	Trails /Day	Time /Trail
Test 1	*Ddb1*^*F/F*^	5 males and 3 females / 5–8 month	10 cm	3	90s	3	60s
	*Ddb1*^*F/F*^*; Camk2α-Cre*	4 males and 3 females / 5–8 month					
Test 2	*Crbn*^*F/F*^	2 males and 3 females / 7–9 month	10 cm	4	60s	4	60s
	*Crbn*^*F/F*^*; Nestin-Cre*	4 males and 5 females / 7–9 month					
Test 3	*Crbn*^*F/F*^	1 male and 7 females / 8–12 month	12.5 cm	4	90s	4	90s
	*Crbn*^*F/F*^*; Nestin-Cre*	2 males and 5 females / 9–10 month					
	*Crbn*^*F/F*^*; Nestin-Cre +* BMS-204352	2 males and 5 females / 9–10 month					

### Drug administration

Patched cells were perfused with the bath solution containing 10 μM BMS-204352 and used to detect the whole cell currents. For mouse work, BMS-204352 was dissolved in the vehicle solution (DMSO 1/80; Tween 80 1/80; 0.9% NaCl) and administered with a 10 ml/kg single intraperitoneal (i.p) injection. In the test 3 of Morris water maze, mouse was pretreated with vehicle or BMS-204352 (2mg/kg) during every training days. Behavioral tests were performed at the maximal BMS-204352 brain concentration, i.e., 30 min after injection [40].

### cDNA and plasmids

The full-length cDNAs (Human ORFeome Collection, MA) of *CRBN* and *FBXO7* were amplified and subcloned into pXF4H expression vector (2xHA in the N-terminus) from Xin-Hua Feng (Zhejiang University, China) between the HindIII and XbaI restriction sites, and named as HA-CRBN and HA-Fbxo7. The full-length of *SLO1* and *FBXO7* were amplified and subcloned into pXF6F expression vector (3xFlag in the N-terminus) from Xin-hua Feng between the BamHI and EcoRI restriction sites, and named as Flag-Slo1 and Flag-Fbxo7. The full-length of *CUL1* and *SKP1* were amplified and subcloned into pXF3HM expression vector (His-6xMyc in the N-terminus) from Xin-hua Feng between the HindIII and XbaI restriction sites, and named as Myc-Cul1 and Myc-Skp1.

### RNAi

The siRNAs were synthesized by Ribobio Company (Guangzhou, China) and transfected using lipofectamine RNAiMax, Lipofectamine 2000 or Lipofectamine 3000 (Invitrogen). Protein were harvested at 48h after transfection. The sequences of siRNA are listed as follows: (**Table 2**)

**Table 2:** siRNA sequences.

siDDB1	5’-ACUAGAUCGCGAUAAUAAAdTdT-3’
	5’-CCUGUUGAUUGCCAAAAACdTdT-3’
siCul4A	5’-GCAAAGCAUGUGGAUUCAAAGUUAA-3’
siCul4B	5’-AAGCCUAAAUUACCAGAAAdTdT-3’
siCRBN	5’-CAGCUUAUGUGAAUCCUCAUGGAUA-3’
siFbxo7	5’-CCCACACCAUUCCAUUCUA-3’
siCul1	5’-GUUCAUAGCAGCCAGCCUGdTdT-3’
	5’-GGCUUGUGGUCGCUUCAU-3’

### Western blot

Cultured cell samples were lysed in RIPA buffer (Sigma-Aldrich) or NETN buffer (150 mM NaCl, 1% NP-40, 50 mM Tris–HCl, pH 8.0) supplemented with complete set of protease inhibitors (Roche) for 20 min in ice. After centrifugation at 17,950 rpm for 15 min, supernatants were collected and subjected to protein quant (Pierce BCA Protein Assay Kit, Thermo). Mouse brain samples were homogenized in the same RIPA buffer, followed by incubation on ice for 30 min, and centrifuged for 15 min at 12,000 g, 4°C to collect proteins in supernatants. Equal amounts from 10–100 μg of proteins were separated by SDS-PAGE and transferred onto PVDF or nylon membranes (Invitrogen). After blocking in 5% non-fat dry milk and incubation with indicated primary antibodies in 5% BSA at 4°C overnight followed by HRP-linked secondary antibodies for 1 hour, bands on membranes were detected using chemiluminescent substrates (Thermo Scientific).

### Co-immunoprecipitation

For transient transfection, 293T cells were seeded at 2 x 10^6^ cells per dish (60 mm diameter). On the next day, expression constructs (usually 1 μg for each construct, total constructs ≤ 5 μg) were introduced into cells (70–90% confluent at transfection) using Lipofectamine 2000 or Lipofectamine 3000. 48 hr later, Cells were lysed in RIPA buffer or NETN buffer supplemented with complete protease inhibitors cocktail. After centrifugation at 17,950 rpm for 15 minutes, supernatants were subjected to incubation with anti-Flag M2 Affinity agarose or anti-HA Affinity agarose overnight at 4°C. After washing 3 times with lysis buffer, beads were added with SDS sample buffer, boiled and analyzed by Western blot.

### Ubiquitination assay

To detect ubiquitination status of protein, 293T cells co-transfected with indicated plasmids were lysed after treatment with MG132 (10 μM) for 6h. Lysates were subjected to co-immunoprecipitation and analyzed by Western blot using indicated antibodies.

### CRISPR genome editing

The genome editing plasmids were prepared by cloning sgRNAs into pX459 plasmid (Addgene) digested by BbsI (NEB) following Zhang’s lab protocol (http://www.addgene.org/crispr/zhang/). 293T, U87mG and LN229 cells cultured in 6-well plates were transiently transfected with 2 μg of pX459 genome editing plasmids using Lipofectamine 3000 (Invitrogen). Two days after transfection, cells were treated with growth media containing puromycin (0.6–1 μg/mL) for 3 days. After recovery in complete media for 2 days, cells were collected to check the *SLO1* editing by Western blot. Cells for *CRBN* editing were transferred into 96-well plate via serial dilution for single cloning (0.3 cells/well). Single clones were screened and expanded to investigate CRBN editing. sgRNA sequences: (**Table 3**)

**Table 3:** sgRNA sequences for gene editing.

sgCRBN-1	Forward 5’-CACCGCAGGACGCTGCGCACAACA-3’
	Reverse 5’-AAACTGTTGTGCGCAGCGTCCTGC-3’
sgCRBN-2	Forward 5’-CACCGTCCTGCTGATCTCCTTCGC-3’
	Reverse 5’-AAACGCGAAGGAGATCAGCAGGAC-3’
sgSlo1	Forward 5’-CACCGATGATGAGCGCATCCATCT-3’
	Reverse 5’-AAACAGATGGATGCGCTCATCATC-3’

### Membrane protein extraction

Integral membrane proteins and membrane-associated proteins were enriched using the Thermo Scientific Mem-PER Plus Membrane Protein Extraction Kit (89842) following manufacture’s protocol.

### Lentivirual vector

WT and R419X *CRBN* sequences were subcloned into pcDNA3-YFP vector. Then YFP-CRBN (WT and R419X) sequences were subcloned into pLVX-IRES-Neo vectors (Clontech). The cloned pLVX vectors were transfected into 293FT cells along with psPAX2 and pVSVg using Lipofectamine 2000 (Invitrogen) for virus packaging. The viral supernatant was collected 72 hours later and condensed by ultra-centrifugation at 25,000 rpm at 4°C for 2 hours. *CRBN* KO U87mG cells were treated with 6 μg/ml Polybrene (Sigma) during the viral infection (MOI = 0.5). Two day after infection, cells were selected with 500 μg/mL G418 for 2 weeks. Stable cells were transferred into 96-well plates with limiting dilution. Single clones were expanded and collected for immunoblot analysis.

### Q-PCR

RNA was extracted with trizol regent (Invitrogen) and reverse transcripted using PrimeScript RT Master Mix (Takara, RR036A). Relative expression level of Slo1 transcripts was determined by real time RT-PCR using SYBR Green (Life Technology) with GAPDH as internal control. Primers are listed below: F: GGCAGCAGTCTTAGAATGAGTAG; R: AAAGCCCACCACATGCGTT.

### Patch clamping

Signals were recorded with an Axopatch 200B amplifier (Axon Instruments). Conventional whole-cell and inside-out configurations of the patch-clamp technique were used in the electrophysiological study. Records were filtered at 2 kHz and digitized at 20 kHz. Patch electrodes were pulled from a horizontal micropipette puller (P-1000, Sutter Instruments) and flame polished to final tip resistance of 4–6 MΩ when filled with internal solutions. The pipette solution (130 mM potassium aspartate (Sigma-Aldrich), 10mM KCl, 1 mM MgCl_2_, 10 mM HEPES, 5 mM EGTA at pH 7.3 (adjusted with KOH)) and bath solution contained (140 mM NaCl, 5.4 mM KCl, 1.8 mM CaCl_2_, 1 mM MgCl_2_, 10 mM HEPES, 10 mM glucose at pH 7.4 (adjusted with NaOH)) were used for whole-cell recordings. For single-channel recordings, the pipette solution (extracellular) contained 120 mM KCl, 20 mM KOH, 11 mM EGTA and 10 mM HEPES (pH 7.4 with KOH), and the bath solution (intracellular) contained 120 mM KCl, 20 mM KOH, 11 mM EGTA, 1 mM MgCl_2_, and 10 mM HEPES (pH 7.4 with KOH). All of the experiments were performed at room temperature (22± 1°C). Data acquisition and analysis were carried out using pClamp 10.2 (Axon Instruments). Records were filtered at 2 kHz and digitized at 20 kHz. The whole-cell currents were elicited from a 0 mV holding potential to test potentials from −100 to +100mV in 10 mV increments (see inset). The BK currents were paxalline-subtracted currents and normalized to membrane capacitance. Single-channel events were analyzed by pclampfit 10.2 (single-channel search-in-analyze function). NPo, the product of the number of channels and open probability, was used to measure channel activity within a patch. In total, 50% threshold cross-method was used to determine valid channel openings.

### Statistical analysis

Results were presented as mean±standard error of mean (SEM). Results for different groups were compared by the two-tailed unpaired Student’s *t* test with Welch’s correction. Results for Barnes maze, locomotor activity test and training days of Morris water maze were performed with two-way repeated-measures ANOVA tests. Asterisks in the Figures were used to indicate statistically significant differences (*P < 0.05; **P < 0.01;***P < 0.001).

## Supporting information

S1 Fig Behavior analysis in DDB1 deficient mice, related to Fig 1. All behavior analysis data are shown as mean ± SEM.
(A) One hour test of locomotor activity yielded no significant differences between the genotypes (*Ddb1*^*F/F*^ *n* = 16, *Ddb1*^*F/F*^*;Camk2a-Cre n* = 15, no sex effect, nor genotype*sex interaction) in ambulation (*F*_*(1*,*29)*_ = 0.01268, *P* = 0.911), rearing (*F*_*(1*,*29)*_ = 0.642, *P* = 0.4297), center activity (*F*_*(1*,*29)*_ = 1.933, *P* = 0.175) or total horizontal activity (*F*_*(1*,*29)*_ = 0.007, *P* = 0.934).
(B) Light/Dark transfer to assess anxiety-like behavior in mice. There was no significant difference between *Ddb1*^*F/F*^ mice (*n* = 16) and *Ddb1*^*F/F*^*;Camk2a-Cre* mice (*n* = 15) in their time spent in the light compartment (*P* = 0.423). But *Ddb1*^*F/F*^*;Camk2a-Cre* mice exhibited less light/dark compartment transitions than *Ddb1*^*F/F*^ mice (*P* = 0.042).
(C) Vision testing. In a visual cliff test, both *Ddb1*^*F/+*^*;Camk2a-Cre* (*n* = 5) and *Ddb1*^*F/F*^*;Camk2a-Cre* (*n* = 5) mice were able to avoid stepping down onto the Plexiglas surface of the visual cliff and exhibited no significant differences (*P* = 0.837).
(TIFF)

S2 Fig Cued and contextual fear conditioning test in DDB1 and CRBN deficient mice, related to Fig 2. All behavior analysis data are shown as mean ± SEM.
(A) Cued and contextual fear conditioning test of *Ddb1*^*F/F*^ (*n* = 15) versus *Ddb1*^*F/F*^*;Camk2a-Cre* (*n* = 16) mice. The mean freezing time during the cue exposure trial was significantly different between the genotypes (*P* = 0.0485).
(B) Cued and contextual fear conditioning test of *Crbn*^*F/F*^ (*n* = 9) versus *Crbn*^*F/F*^*;Nestin-Cre* (*n* = 9) mice. The mean freezing time during the cue exposure trial was significantly different between the two genotypes (*P* = 0.0142).
(TIFF)

S3 Fig DDB1-binding-defective CRBN is targeted by SCF^Fbxo7^ E3 ligase, related to Fig 3. (A) Human intellectual disability associated CRBN^R419X^ mutant (marked in orange) causes a premature stop codon after Arg 419, resulting in a truncated CRBN loss of several residues. These residues have very intensive interactions (indicated in yellow dash) with its adjacent parts of CRBN (highlighted in sticks), which could probably disrupt the required interaction of CRBN structure, and thus cause CRBN losing its normal conformation for DDB1 binding.
(B) Western blot analysis for protein levels of WT and mutant CRBN after treatment with MG132 (20 μM) for 8 hr or not.
(C) CHX (100 μg/ml) chasing assays for the stability of CRBN and CRBN^R419X^ (Top). Quantification of the CRBN^R419X^ protein level was normalized to actin and relative to the normalized CRBN^R419X^ at the 0 hr time point (Bottom).
(D) CHX (100 μg/ml) chasing assays for the stability of CRBN and CRBN^D249Y^ (Top). Quantification of the CRBN^D249Y^ protein level was normalized to actin and relative to the normalized CRBN^D249Y^ at the 0 hr time point (Bottom).
(E) Western blot analysis for protein levels of mutant CRBN in 293T cells treated with MLN4924 (0.1μM) for indicated time.
(F) Co-immunoprecipitation of CRBN with different Cullin proteins that were over-expressed in 293T cells.
(G) Co-immunoprecipitation of WT or mutant HA-CRBN with Myc-Cul1 in 293T cells after treatment with MG132 (10 μM) or non-treatment.
(H) Co-immunoprecipitation of WT or mutant HA-CRBN with Flag-Fbxo7 after overexpression in 293T cells.
(I) Western blot analysis to detect the levels of ubiquitinated WT or mutant HA-CRBN in 293T cells overexpressing Flag-Fbxo7 and Myc-Cul1 alone or in combination. Cells were treated with MG132 (10 μM) for 6 hrs before protein extraction.
(J) Western blot analysis to detect the levels of ubiquitinated HA-CRBN^R419X^ in 293T cells. Cells overexpressing Myc-Fbxo7 alone were transfected with siRNA for GFP or Fbxo7 and treated with MG132 (10 μM) for 6 hrs before protein extraction.
(TIFF)

S4 Fig Disruption of CRL4^CRBN^ destabilizes Slo1, related to Fig 4. (A) Quantification of Slo1 levels (detected by anti-Slo1, Novus) in brain extracts isolated from WT (*n* = 3) and *Ddb1*^*F/F*^*;Camk2a-Cre* (*n* = 4) mice. Protein levels were standardized as the ratio of Slo1 signals to the cognate actin signals, followed by normalization to the mean value of standardized Slo1 signals in WT mice. Data are shown as mean ± SEM. *P* < 0.05.
(B) Western blot analysis for Slo1 levels in brain extracts isolated from WT and *Crbn*^*F/F*^*;Nestin-Cre* mice with the L6/60 monoclonal anti-mSlo1 antibody (Millipore). Quantification of Slo1 level was standardized as the ratio of Slo1 signals to the cognate beta-actin signals, followed by normalization to the mean value of Slo1 signals in WT mice. Data are shown as mean ± SEM. *P* < 0.05.
(C) Western blot analysis for Slo1 levels in 293T cells transfected with Flag-Slo1 along with indicated siRNAs.
(D) Western blot analysis of Slo1 levels in indicated cells. U87mG and LN229 cells were transfected with sgCtrl (-), sgSlo1 (+) or not (WT).
(E) Western blot analysis of CRBN levels in single clones of 293T cells edited by CRISPR/Cas9 system for CRBN deletion.
(F) CHX (100 μg/ml) chasing assays for the stability of Slo1 in *CRBN* KO 293T expressing WT or mutant CRBN. Plot shows quantification of Slo1 normalized to actin and relative to the normalized Slo1 at the 0 hr time point.
(G) Western blot analysis of CRBN or Slo1 levels in single clones of U87mG glioma cells edited by CRISPR/Cas9 system for CRBN deletion.
(H) Western blot analysis of Slo1 levels in cytoplasm or membrane extracts of WT or *CRBN* KO LN229 cells.
(TIFF)

S5 Fig Disruption of CRL4^CRBN^ redirects Slo1 to SCF^Fbxo7^ for degradation, related to Fig 5. (A) Q-PCR analysis of levels of Slo1 mRNA expression between WT and *CRBN* KO glioma cells. Data are shown as mean ± SEM and represent three biological replicates.
(B) Co-immunoprecipitation of Slo1 with WT or mutant CRBN and Fbxo7 in *CRBN* KO 293T cells. Cells were transfected with expression vectors encoding indicated genes followed by IP with Flag affinity beads.
(C) Co-immunoprecipitation of Flag-Slo1 with Myc-Cul1 in *CRBN* KO 293T cells overexpressing HA-Fbxo7 and empty HA or WT/R419X CRBN.
(D) Co-immunoprecipitation of truncated Slo1 with Fbxo7 in *CRBN* KO 293T cells.
(TIFF)

S6 Fig Patch Clamp in CRBN KO LN229 cells, related to Fig 6. (A) The whole cell currents of LN229 cells were detected by voltage clamp. BK currents were presented as the difference between the currents under basal condition and after treatment of Paxilline (10 μM, BK channel blocker) for at least 10 mins. The I-V curve of BK currents in WT and *CRBN* KO cells were shown on the right panel (*n* = 5 for each group, *P* < 0.01).
(B) The whole cell currents of U87mG cells were detected by voltage clamp. The I-V curve of BK currents (paxalline-subtracted currents) in WT cells and WT cells treated with MLN4924 (0.1μM) for 24 hrs were shown on the right panel (n = 5 for each group).
(TIFF)
